# Supplementary figures and images for: Lost in HELLS: Disentangling the mystery of SALNR existence in senescence cellular models
Source: PLoS One. 2023 May 30;18(5):e0286104. doi: 10.1371/journal.pone.0286104 (PMC10228806; doi:10.1371/journal.pone.0286104)

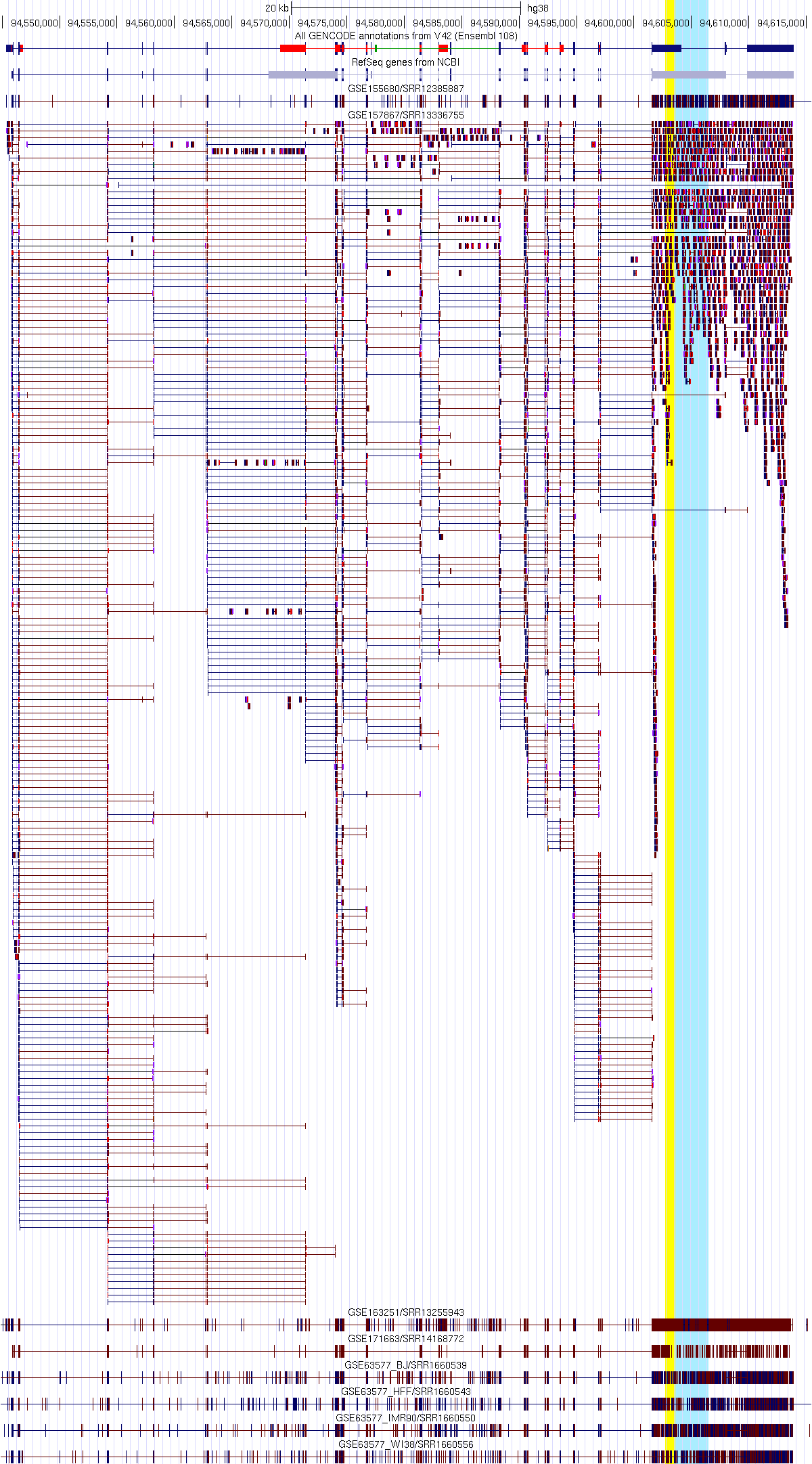

Supplement: S1 Fig — Visualization of sequence reads (from short-read RNA-seq) aligned to the genomic portion including HELLS and SALNR, using UCSC genome browser. It is not possible to isolate SALNR expression from this type of reads, but the heavy presence of reads covering the 3’ end of the HELLS gene suggests an interesting production of known and novel isoforms in that location. This figure represents the sample with highest read count for each dataset (from proliferative cells). The GSE157867 track (the dataset with 2x150bp reads) is plotted in “squish” display mode, while the others are plotted in “dense” mode. The short-read RNA-seq mapping tracks can be visualized on UCSC genome browser: https://genome.ucsc.edu/s/cnr.itb.ba/short-read_RNA-seq. See Data Availability section. (TIF) [file pone.0286104.s001.tif]

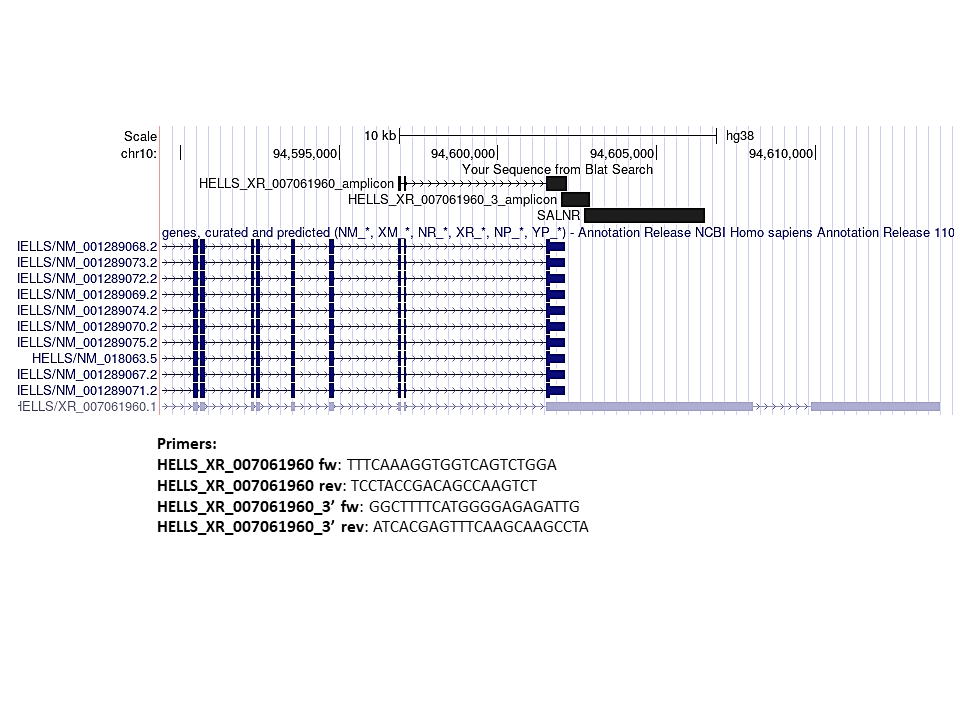

Supplement: S2 Fig — Genomic location of the two amplicons used to amplify the XR_007061960 isoform of HELLS and corresponding primer sequences. (TIF) [file pone.0286104.s002.tif]

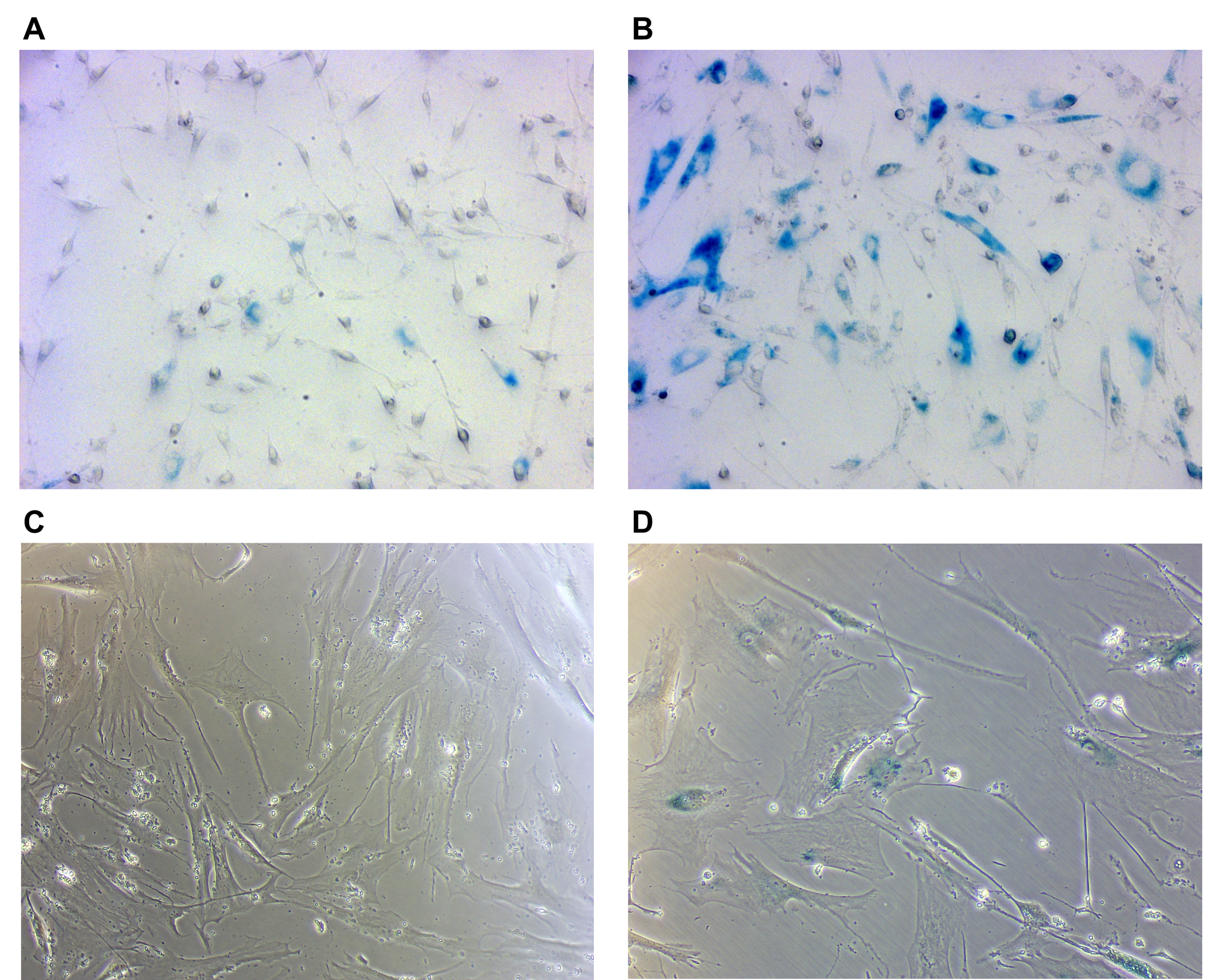

Supplement: S3 Fig — Representative images of colorimetric β-gal assay in young (p6) (A) and old (p16) (B) HMSCs, and in young (p6) (C) and old (p16) (D) NHDFs (x10 magnification). (TIF) [file pone.0286104.s003.tif]
