# Supplementary material for: Lost in HELLS: Disentangling the mystery of SALNR existence in senescence cellular models
Source: PLoS One. 2023 May 30;18(5):e0286104. doi: 10.1371/journal.pone.0286104 (PMC10228806; doi:10.1371/journal.pone.0286104)
Supplement: S1 Raw images — (PDF) [file pone.0286104.s009.pdf]

IMAGES CAPTURED WITH

Imaging system: Uvitec Essential V6 17.06 (Cambridge)

Software: Uvitec 1D

KEY

M. MW Marker, 1. Bladder, 2. Brain, 3. Cervix, 4. Esophagus, 5. Kidney, 6. Liver, 7. Lung, 8. Ovary, 9. Skeletal muscle, 10. Small intestine, 11. Spleen, 12. Testis.

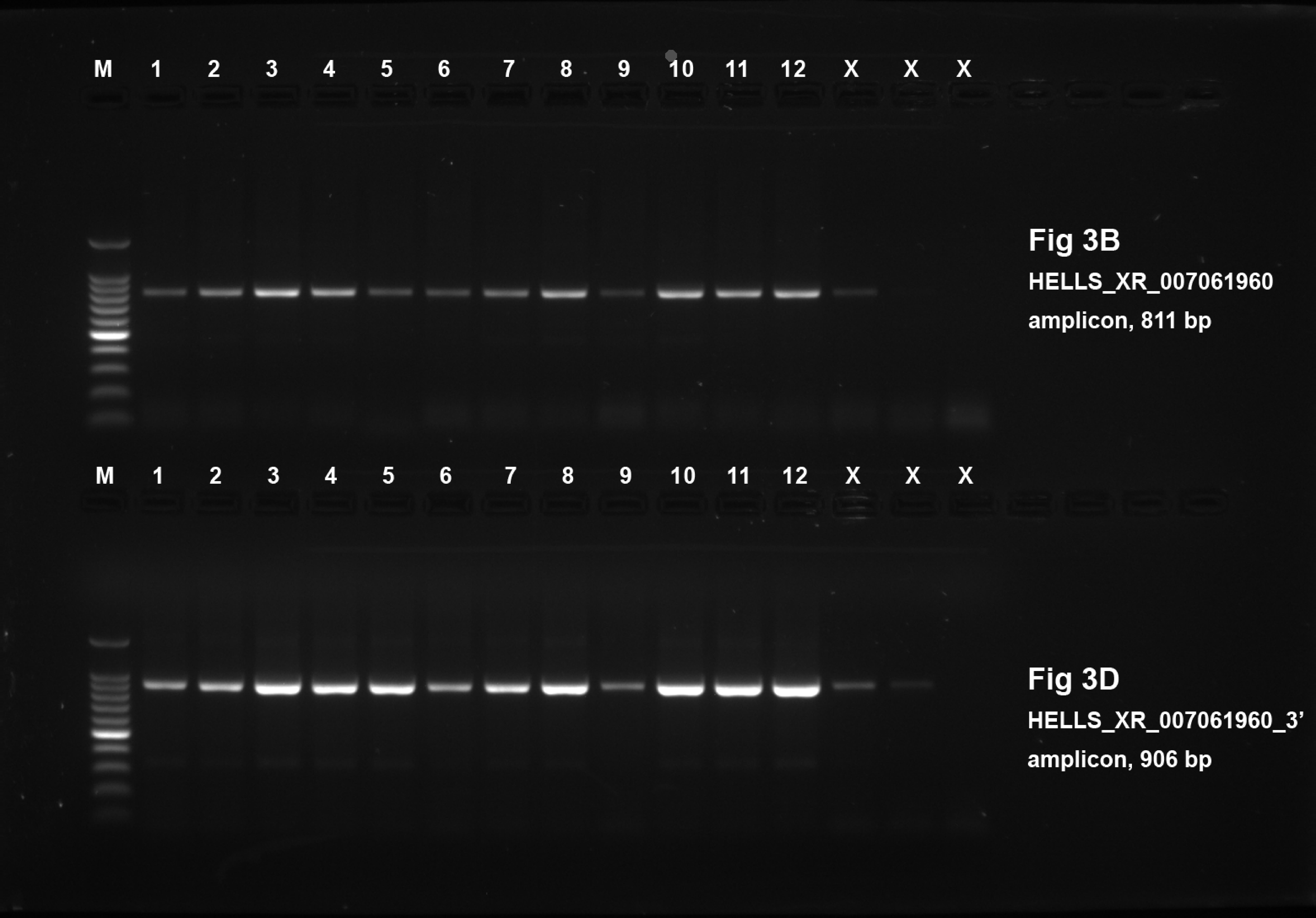

IMAGES CAPTURED WITH
Imaging system: Uvitec Essential V6 17.06 (Cambridge)
Software: Uvitec 1D

KEY
M. MW Marker, 1. SH-SY5Y,
2. HSMC, 3. NHDF, 4. HUVEC

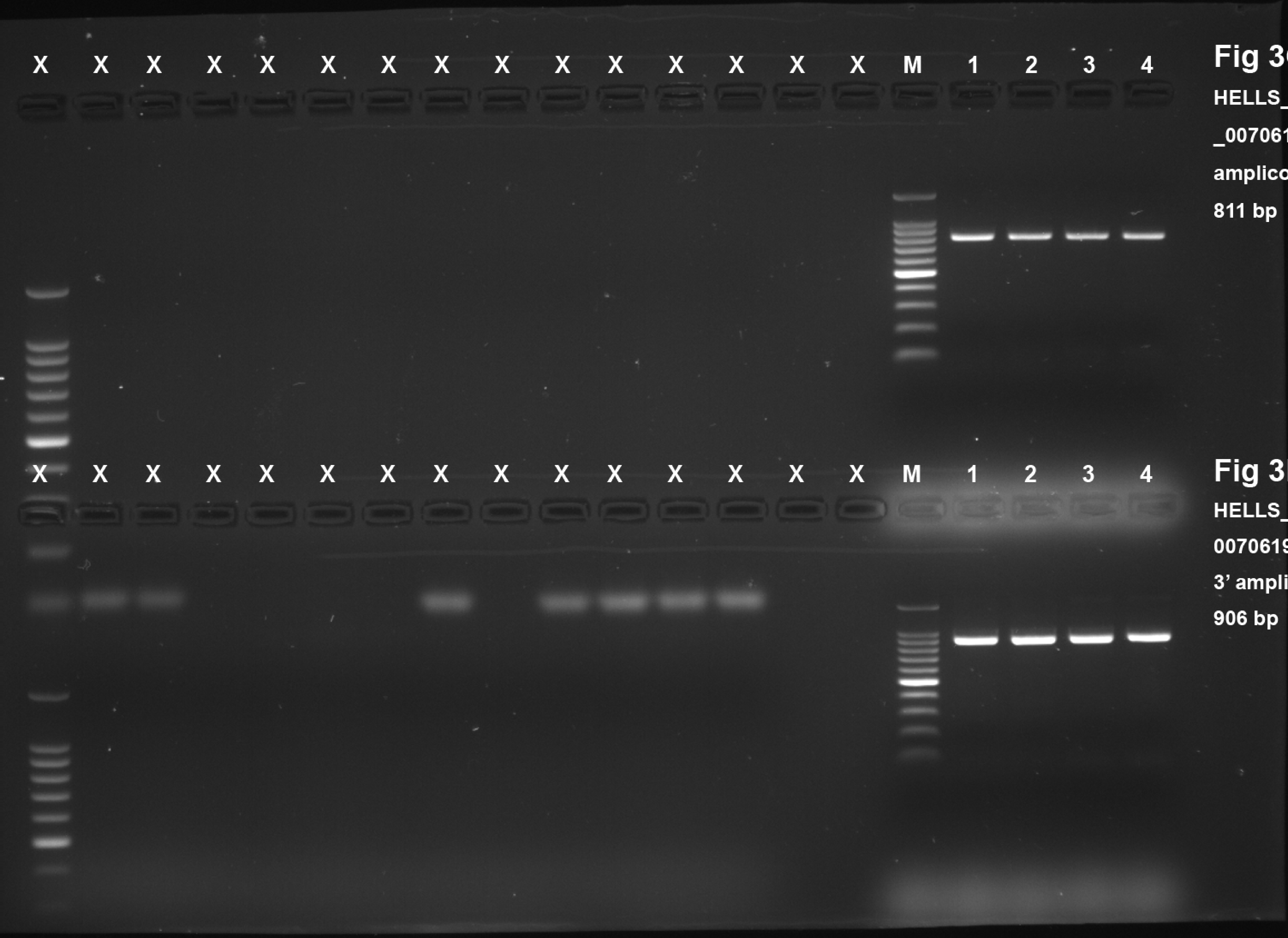

Fig 3C
HELLS_XR_007061960 amplicon, 811 bp

Fig 3E
HELLS_XR_007061960_3' amplicon, 906 bp
